# Strengthening tobacco control research: key factors impacting policy outcomes and health equity

**DOI:** 10.3389/fpubh.2024.1501326

**Published:** 2024-12-20

**Authors:** Bukola Usidame Peters, Natalie McArthur, Andrea Titus

**Affiliations:** ^1^Department of Public Health, Purdue University, West Lafayette, IN, United States; ^2^NYU Grossman School of Medicine, New York, NY, United States

**Keywords:** smoke-free, policy, tobacco, smoking, Tobacco Nation

## Abstract

In this policy brief, we explore several potential drivers of heterogeneity in policy outcomes that can be examined in tobacco control policy evaluations, expanding the evidence base to contribute to continued, equitable progress in reducing tobacco-related health outcomes. We discuss these factors in the context of a hypothetical evaluation of the impact of smoke-free laws on current smoking and quit attempts in the Tobacco Nation. Despite a similar policy environment within the Tobacco Nation, there is variation in the strength of smoke-free law coverage across states. This commentary considers how policy design and other contextual factors, including co-occurring policies, and differential impacts across subgroups, may influence policy-attributable outcomes across time and space.

## Introduction

The United States (U.S.) has made substantial gains in recent decades in reducing tobacco use and associated health outcomes. This progress has occurred in the absence of strong federal tobacco control laws. States and localities across the U.S. have implemented tobacco control legislation to protect their communities from the dangers of tobacco use and promote public health benefits. However, substantial geographic and sociodemographic disparities in tobacco use impede equal progress. These disparities stem in part from uneven coverage by tobacco control policies across time and space, as some states/localities have particularly strong tobacco control environments, while others have lagged (1). Similar policies may also lead to different outcomes across different contexts. For example, while a number of studies have found that smoke-free laws are associated with public health benefits (2) – including reduced exposure to second-hand smoke, increased smoking cessation, decreased smoking initiation, decreased smoking quantity, and decreased current smoking among adults (3) – findings are not consistent. An interrupted time-series analysis comparing impacts of smoke-free laws across multiple locations found no evidence of changes in trends of smoking prevalence after policy introduction in 13 out of 21 jurisdictions included in the study (4).

The fragmented tobacco control policy landscape creates opportunities to evaluate policy impacts using natural experiment study designs. While there have been many tobacco control policy evaluations, there are also gaps in comprehensively understanding the impacts of tobacco control policies. These gaps intersect with increasingly complex regulatory environments, emerging tobacco products, and widening tobacco-related health disparities, among other factors. In this policy brief, we explore several potential drivers of heterogeneity in policy outcomes that can be examined in tobacco control policy evaluations, expanding the evidence base to contribute to continued, equitable progress in reducing tobacco-related health outcomes. These drivers include factors related to policy design, co-occurring policies, and heterogeneity in policy outcomes across population subgroups. We discuss these factors in the context of a hypothetical evaluation of smoke-free laws in “Tobacco Nation” (hereafter, TNa), a group of 12 states throughout the U.S. Midwest and South (5). We focus on TNa for two reasons. First, TNa states have higher smoking prevalence compared to the rest of the U.S. (6), underscoring the urgency of understanding how tobacco control policies can contribute to reducing tobacco-related health outcomes in this region. Second, while TNa states have relatively weak tobacco control policy environments overall, there is considerable heterogeneity in smoke-free law coverage across TNa states, which can be examined in a policy evaluation.

## Tobacco control policies within TNa

In 2021, TNa states (Alabama, Arkansas, Indiana, Kentucky, Louisiana, Michigan, Mississippi, Missouri, Ohio, Oklahoma, South Carolina, Tennessee, and West Virginia) had the highest adult smoking prevalence in the country with an average of 17.2%, compared to all other non-TNa states with an adult smoking prevalence of 12.6% (6). TNa states also have weak tobacco control policy environments. The American Lung Association (ALA) collates a report card annually that scores every state’s tobacco policies and provides letter grades, A (excellent) to F (inadequate) based on policy characteristics (7) across five key areas: prevention and cessation funding, excise taxes, access to cessation services, flavor bans and smoke-free air laws. According to the ALA, all but one of the TNa states (Oklahoma) have F letter grades on their overall tobacco policies (7). There are multiple reasons for weak policy environments in TNa, despite high levels of support within the population for specific tobacco control initiatives (8). Some of these reasons include pre-emption laws that prevent local jurisdictions from implementing strong local tobacco control policies, the influence of the tobacco industry, or economic dependence on tobacco farming, or ineffective local coalitions (9).

**Figure 1:**
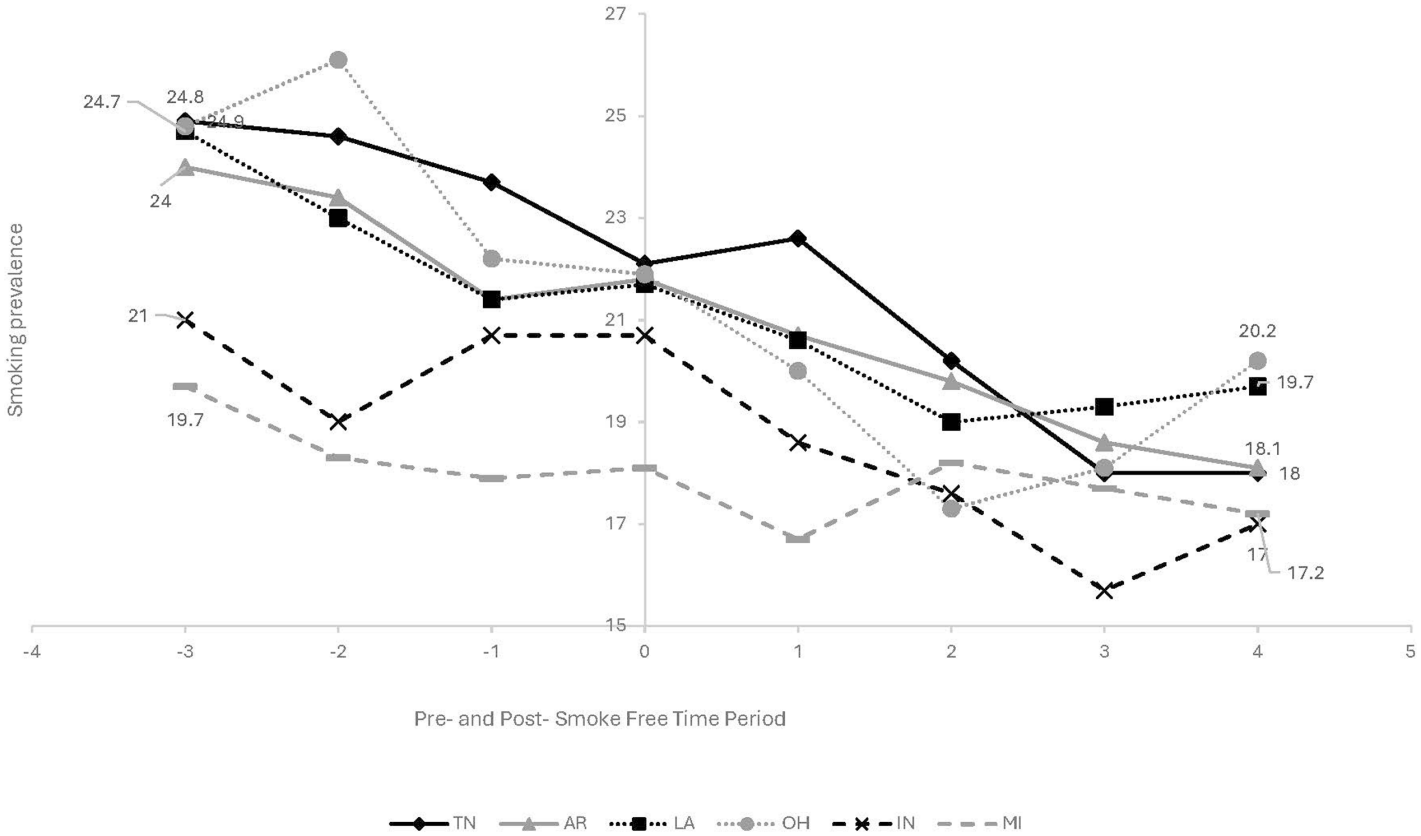
Current smoking yearly trends pre-and post-smoke free law among 6 Tobacco Nation States – Behavioral Risk Factor Surveillance Survey. Year policy passed (0)-OH & AR - 2006, TN & LA - 2007, MI - 2010, IN - 2012; shaded area represents the period the smoke-free law was passed. Each State’s trend is represented by a unique line style (solid, dashed, dotted) and marker type (circle, square, triangle). Data were plotted directly from raw annual prevalence rates for each state, with no regression modeling performed.

**Figure 2:**
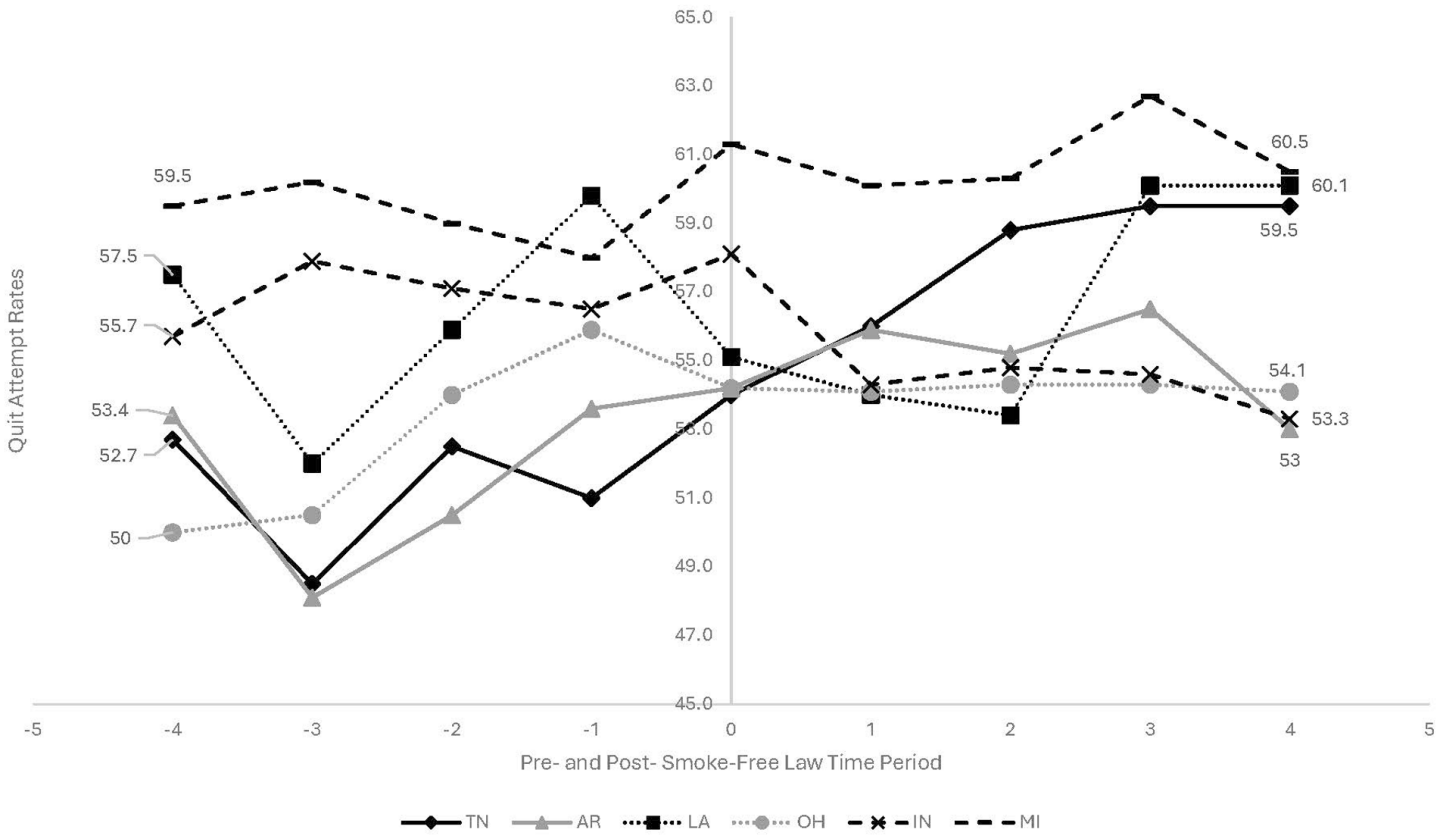
Quit attempt yearly trends pre-and post-smoke free law among 6 Tobacco Nation States – Behavioral Risk Factor Surveillance Survey. Year policy passed (0)-OH & AR - 2006, TN & LA - 2007, MI - 2010, IN - 2012; shaded area represents the period the smoke-free law was passed. Each State’s trend is represented by a unique line style (solid, dashed, dotted) and marker type (circle, square, triangle). Data were plotted directly from raw annual prevalence rates for each state, with no regression modeling performed.

Despite an overall ‘F’ grade in most TNa states, there is considerably more heterogeneity within specific policy domains. For example, six TNa states have implemented comprehensive smoke-free laws, which ban smoking in workplaces, restaurants, bars, and/or other venues. To contextualize smoke-free laws within broader smoking trends within TNa, we analyzed the patterns of smoking prevalence and quit attempt trends pre-and post-smoke-free laws within the TNa states. We focused on describing trends over time, rather than assessing causal impacts or associations with smoke-free laws. Using Behavioral Risk Factor Surveillance Survey (BRFSS) data for all six states with smoke-free laws, we descriptively assessed the yearly weighted prevalence of current smoking (Figure 1) and quit attempts (Figure 2). Current smoking was defined as having smoked 100 or more cigarettes in a respondent’s lifetime and smoking ‘every day’ or ‘some days’ now and quit attempts was defined as having stopped smoking for a day or longer in the past 12 months because the respondent was trying to quit. Data included 3 years pre-policy and 4 years post-policy; OH (2003–2010), IN (2009–2016), AR (2003–2010), TN (2004–2011), MI (2007–2014), and LA (2003–2010). More information on the BFRSS methodology can be found here (10).

There are several takeaways from these figures.

First, in most TNa states, current smoking prevalence was declining and quit attempts were increasing, prior to smoke-free policy adoption. In other words, smoke-free laws were introduced in the context of strong secular trends in combustible tobacco use. Second, despite these overall trends, pre-and post-policy slopes differ across states. The remainder of this policy brief uses this example of smoke-free laws in TNa as a starting point for considering how contextual, policy, and population factors may be incorporated into tobacco control policy evaluations and shed light on drivers of policy effect heterogeneity. We explore three factors: policy design considerations, co-occurring policies, and differential impacts across subgroups.

## Policy design considerations

Individual tobacco control policies may vary considerably from one jurisdiction to another. In addition to grading overall tobacco control policy environments, the ALA also grades the strength of policies within each area (e.g., smoke-free laws), shedding light on policy variation across areas. For example, regarding smoke-free laws, grading is based on how many venues are covered and whether e-cigarettes are included in the policy. There is considerable variation in smoke-free policy design across TNa states. While Ohio boasts one of the most robust smoke-free laws in the nation, earning an A grade, other states—Indiana, Arkansas, Michigan, Tennessee, and Louisiana—have weaker smoke-free laws that fully or partially exclude certain venues or exclude e-cigarettes, earning a weaker grade. For example, TN and AR have only partial bans on smoking in restaurants, as restaurants can allow smoking on outdoor patios (7).

When evaluating tobacco control policies, and particularly when combining information across multiple states or jurisdictions, researchers often must make explicit decisions about which types of variation can reasonably be ignored and which types of variation may be relevant to the outcome being studied. However, there is a relatively limited body of evidence for making these judgments. While some prior research suggests that “comprehensive” tobacco laws (e.g., smoke-free laws that prohibit smoking in all public places and workplaces, some including vape products) (11) have a more significant positive impact on health outcomes compared to partial or nonexistent laws, this finding is not consistent (11, 12). Considering various aspects of policy design may be particularly important in the context of emerging tobacco products. For example, in one recent analysis of smoke-free laws, the authors note that their results “do not rule out” the possibility that adding vaping restrictions to smoke-free workplace laws could modestly attenuate the impacts of these laws on current smoking behavior among emerging adults (11). Future studies that further explore variation in tobacco control policies across jurisdictions may shed additional light on the potential for different dimensions of policy design to be salient across a range of tobacco-related outcomes.

## Co-occurring policies

Individual tobacco control policies are not implemented in a vacuum. Considering other aspects of the policy environment in a policy evaluation is important for two reasons. First, other policies—alongside other sociodemographic and population characteristics—may be important confounders that could bias the estimated effect of a policy on a health outcome if not accounted for in statistical models. Prior research suggests that many social policies, including tobacco control policies, are highly correlated, and that policy evaluations often may not adequately address such policy co-occurrence, in part because accounting for highly correlated policies could lead to decreases in statistical precision (13). However, recent scholarship has also outlined several approaches for addressing this collinearity, including applying Bayesian methods and evaluating policy “clusters” rather than individual policy interventions (14).

Second, co-occurring policies may be important to consider from the perspective of effect modification or statistical interaction. At present, there is very limited and mixed evidence regarding the impact of policy interactions on tobacco outcomes (15). Studies suggest that the potential for positive synergistic effects of policies may depend on the specific outcome being studied. For instance, in a recent study, smoking bans were independently associated with reduced social smoking, while high excise taxes were linked to reduced heavy smoking. However, excise taxes only seemed to influence reduced social smoking in the absence of a smoking ban (16). On the other hand, other research suggests that the odds of adolescent electronic nicotine delivery system (ENDS) use is lower when smoking bans and age-of-purchase laws are both implemented relative to age-of-purchase laws alone (17). Furthermore, communities with stronger tobacco industry denormalization initiatives (commonly introduced through media and educational campaigns) tend to reap greater benefits from individual or multiple tobacco control policies in place (18). A prior study using BRFSS data found that the extent to which tobacco control laws reinforce one another with regard to lowering smoking rates may vary across population subgroups, including by age and race/ethnicity (19). In the context of TNa, since TNa states generally have relatively weak tobacco control environments, evaluating the effects of smoke-free laws in these states may shed light on policy outcomes in places without strong funding for prevention or cessation services, or other types of tobacco control policies.

## Differential impacts across subgroups

Examining the impacts of tobacco control policies on different population groups – particularly groups who have been disproportionately harmed by tobacco – is essential to understanding the impacts of tobacco control policies on health equity. While the TNa region represents a geographic disparity in smoking prevalence, there are disparities in tobacco use within TNa states by urbanicity (20), markers of socioeconomic status (21), and other factors. Increasingly, tobacco control evaluations are examining subgroup variation in policy effects using regression-based strategies, including stratification and interaction models. While these approaches add to our understanding of the impacts of tobacco control policies on health equity, other forms of data collection, including the adoption of implementation science approaches or community-based research principles can further illuminate context-specific factors that influence policy-related outcomes (21). For example, a recent analysis smoke-free policies in public housing developments in New York City paired quantitative and qualitative methods to highlight potential reasons why these policies were not associated with short-term improvements in air quality, including barriers to compliance and enforcement (22). Compliance challenges may extend to other types of smoke-free policies within TNa. A 2007 report from the University of California showed that enforcement for the Ohio smoke-free law started 4 months after the policy implementation. In those 4 months, there were efforts by pro-tobacco interest groups to sow confusion and undermine public support, which subsequently hindered compliance with the law (23). In considering the impacts of policies on health equity, examining subgroup variation and integrating different disciplinary perspectives can shed light on important factors that may drive heterogeneity in policy impacts across groups.

## Actionable recommendations

- Studies that use aggregate data from different states and jurisdictions must carefully determine what types of policy variation can be ignored and which is critical to the outcome being observed.
- Particularly in the context of emerging tobacco/nicotine products, there is a need to focus on different aspects of the policy design, e.g., what products, locations, or age-groups are included or exempted. Future studies that examine the variation in policy designs will provide valuable insight into how different elements of policy design impact a variety of tobacco-related outcomes.
- Examining the effect of tobacco laws in TNa states, given their overall weak policy environments, might reveal more about policy impacts in locations without strong funding for prevention or cessation services, or other types of tobacco control policies.
- As we focus on improving policies to achieve health equity, evaluations should examine subgroup variation and incorporate different disciplinary perspectives to reveal key factors that may drive heterogeneity in policy impacts across groups.

## Conclusion

Using the example of policy environments in TNa as a starting point, this policy brief highlights how policy strength or other contextual factors can play a role in shaping policy-attributable outcomes across time and space. Several of these factors have been relatively overlooked in the empirical literature. Enhancing data collection, incorporating interdisciplinary analysis approaches, and centering equity in tobacco control policy evaluations can add to the evidence base for reducing the harms of tobacco use and addressing persistent tobacco-related health disparities.
